# Welcome to Biophotonics Discovery

**DOI:** 10.1117/1.BIOS.1.1.010101

**Published:** 2024-06-21

**Authors:** Darren Roblyer

## Abstract

The editorial announces the launch of Biophotonics Discovery, providing a window into the first issue and a plans for the year ahead.

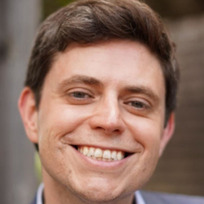



I am delighted to announce the launch of SPIE’s journal *Biophotonics Discovery*. This journal will cover new biological and clinical discoveries enabled by emerging biophotonic technologies, as well as the impact these discoveries are having in translation, basic science, and commercialization. It’s being launched in the recognition that our field has grown substantially over the last several decades and now incorporates physicists, engineers, biologists, physicians, entrepreneurs, representatives from governmental and nonprofit agencies, and others. This diverse and growing community is expanding the scope and applications of biophotonic technologies. These efforts have led to real-world impacts in research and healthcare. Just look at the scientific, commercial, and medical success stories related to OCT, endoscopy, and wearables, among others. In many ways, this journal is a recognition of the successes of our field as measured by the growing influence and impact of our work. Let me further make the case by quoting the former Nobel Laureate Sydney Brenner, who once said: “Progress in science depends on new techniques, new discoveries, and new ideas, probably in that order.”

While a plethora of new techniques and technologies have already been developed and refined by our community, it’s the novel discoveries made using these techniques, along with their impact on both basic science and clinical medicine, that *Biophotonics Discovery* will highlight. The journal will cover topics spanning

• Basic discovery: investigations of new biological observations in cells, organoids, animal models, or human tissue samples using emerging biophotonic technologies
• Physiological monitoring: investigations and advances of physiological signatures in humans and/or preclinical models
• Clinical translation: investigations that involve human measurements in the clinical setting

To emphasize discovery, every published article will include a “Discovery Statement,” which is a short (3 sentences or less) statement that describes the specific scientific discovery and/or the translational impact presented in the article. We have also implemented several novel and defining features, which I summarize here:

• **A progressive open data policy**. Every research publication will include, at least, a so-called “Minimal Data Set,” which includes all the values shown on the figures, and example images when appropriate. This is to encourage rigor, but just as important, it will allow researchers to better utilize the data produced by our community, accelerating discovery.
• **Early career editorial board**. Our early career editorial board includes postdocs and instructors eager to be part of the publishing world. They serve as advisors and as a reviewer pool for the journal.
• **Term limits**. All members of the editorial board have term limits, including myself. This is to provide opportunities for new members of our community to get involved in publishing, and to keep the journal fresh.
• **BIOS Advisory Editors**. This group is composed of the program chairs of the SPIE Photonics West Conference. These editors will help us identify the most exciting work presented at the conference each year, providing inspiration for special sections, editorials, invited research papers, and others.
• **Gold Open Access.** All papers will be free to read and download under a CC-BY 4.0 license, and there will be no publication charges for the first two years.

Submissions to the journal have been brisk, with an impressive quality and breadth. We have just released our first issue, which features four articles highlighting research from Jennifer Barton’s group at the University of Arizona, Melissa Skala’s group at the Morgridge Institute, and Narasimhan Rajaram at the University of Arkansas.

We have several special sections and projects planned. A special section on Optics for Cancer Therapy Monitoring and Diagnosis will be guest edited by Javier A. Jo, Narasimhan Rajaram, Srivalleesha Mallidi, and Alex Walsh. A special section on Skin Tone in Biophotonics will highlight papers that describe how differences in skin tone affect a wide range of optical techniques. Guest editors include Joshua Pfefer (FDA), Jessica Ramella-Roman, Kimani Toussaint, and Matthew Keller. We are planning a special section on Optics for Global Health that will highlight how biophotonics is impacting diverse communities across the globe. Rebecca Richards-Kortum, Vanderlei S. Bagnato, and Jenna Mueller will guest edit this special section. Finally, we are planning a community publication on commercialization of biophotonic technologies. I will spearhead this effort with Gabriela Apiou.

I encourage members of our community to get involved with the journal by submitting manuscripts, suggesting special sections, editorials, reviews, and perspectives, and nominating early-career editors.

I am exceedingly grateful to the wide support I’ve received to start this journal. This includes many leading lights of the community who serve as senior advisors, deputy editors, and associate editors. I’m extremely grateful to the SPIE staff, including Gwen Weerts, Renae Keep, Eva Scalzo, and Patrick Franzen, who have been working tirelessly to get the journal up and running. A special thanks to Anita Mahadevan-Jansen, who supported the journal since the beginning and has been helping me on a week-to-week basis with ideation and strategy. And finally, special thanks to Brian Pogue, Bruce Tromberg, David Boas, and Anna Devor, whose support and discussion has helped refine the vision for this journal.

